# The Effect of Secretory Factors of Adipose-Derived Stem Cells on Human Keratinocytes

**DOI:** 10.3390/ijms13011239

**Published:** 2012-01-23

**Authors:** Kyoung Mi Moon, Ye-Hyoung Park, Jae Seol Lee, Yong-Byung Chae, Moon-Moo Kim, Dong-Soo Kim, Byung-Woo Kim, Soo-Wan Nam, Jong-Hwan Lee

**Affiliations:** 1 Department of Biomaterial Control, Dong-Eui University, Busan 614-714, Korea; E-Mails: omkksm@hanmail.net (K.M.M.); conver100@naver.com (J.S.L.); swnam@deu.ac.kr (S.-W.N.); 2 Prostemics Research Institute, Seoul 135-816, Korea; E-Mails: dpgud04@hanmail.net (Y.-H.P.); dr-dskim@hanmail.net (D.-S.K.); 3 Department of Chemistry, Dong-Eui University, Busan 614-714, Korea; E-Mails: mercenary415@naver.com (Y.-B.C.); mmkim@deu.ac.kr (M.-M.K.); 4 Blue-Bio Regional Innovation Center, Dong-Eui University, Busan 614-714, Korea; E-Mail: bwkim@deu.ac.kr; 5 Department of Biotechnology and Bioengineering, Dong-Eui University, Busan 614-714, Korea

**Keywords:** ADSC-CM, AAPE, stress fiber formation, RhoA-ROCK signaling, regeneration, proliferation, migration

## Abstract

The beneficial effects of adipose-derived stem cell conditioned medium (ADSC-CM) on skin regeneration have been reported. Although the mechanism of how ADSC-CM promotes skin regeneration is unclear, ADSC-CM contained various growth factors and it is an excellent raw material for skin treatment. ADSC-CM produced in a hypoxia condition of ADSC—in other words, Advanced Adipose-Derived Stem cell Protein Extract (AAPE)—has great merits for skin regeneration. In this study, human primary keratinocytes (HKs), which play fundamental roles in skin tissue, was used to examine how AAPE affects HK. HK proliferation was significantly higher in the experimental group (1.22 μg/mL) than in the control group. DNA gene chip demonstrated that AAPE in keratinocytes (*p* < 0.05) notably affected expression of 290 identified transcripts, which were associated with cell proliferation, cycle and migration. More keratinocyte wound healing and migration was shown in the experimental group (1.22 μg/mL). AAPE treatment significantly stimulated stress fiber formation, which was linked to the RhoA-ROCK pathway. We identified 48 protein spots in 2-D gel analysis and selected proteins were divided into 64% collagen components and 30% non-collagen components as shown by the MALDI-TOF analysis. Antibody array results contained growth factor/cytokine such as HGF, FGF-1, G-CSF, GM-CSF, IL-6, VEGF, and TGF-β3 differing from that shown by 2-D analysis. Conclusion: AAPE activates HK proliferation and migration. These results highlight the potential of the topical application of AAPE in the treatment of skin regeneration.

## 1. Introduction

Human subcutaneous adipose tissue contains adipose-derived stem cells (ADSC), which are a population of pluripotent mesenchymal cells and possess the ability to differentiate into various lineages. Some clinical applicabilities of ADSCs that might be of value in compensating defects in damaged neighboring cells have been suggested [1,2]. ADSCs secrete various growth factors that repair and replace the defective surrounding cells. Variously secreted growth factors are a good raw material for skin regeneration, re-epithelialization, wound healing, and wrinkling care instead of ADSC itself. The conditioned medium from ADSCs (ADSC-CM) contained several growth factors secreted from ADSC [3] and has great merit for treatment of skin problems such as wound repair, replacement and regeneration. Recently, ADSCs were isolated from adipose tissue samples via elective liposuction and were cultured in bulk cell factories by our group [4]. ADSC-CM can be applied for biotechnology such as cosmetic skin care products and in the protein drug industries. In this study, we focused on Advanced Adipose-Derived Stem Cell Protein Extract (AAPE), which is a conditioned medium cultured under a hypoxia of adipose-derived stem cells obtained from our group.

Human keratinocytes (HK) play an important role in skin biology such as wound re-epithelialization, and the re-establishment and wound healing of the skin [5–7]. Keratinocytes with normal dermal fibroblasts leads to upregulation of mRNA for collagen type I and III, increased fibroblast proliferation, and extracellular matrix accumulation [8]. Thus, the ability of keratinocyte proliferation and migration is essential for performing these processes on the skin surface. However, no research has reported the biological function of AAPE in HKs, which are major cells in the epithelia. In this study, we examined the effects of AAPE on HK *in vitro*, and the components of AAPE through proteome and antibody array analysis.

## 2. Results and Discussion

### 2.1. HK Proliferation

**Figure 1:**
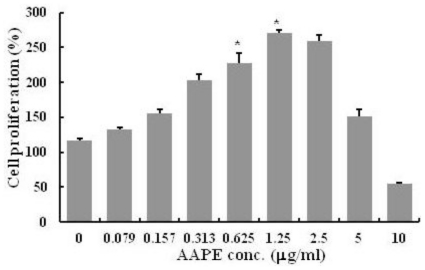
Human Keratinocyte (HK) proliferation. The amount of HK keratinocyte is represented by the cell proliferation (%) in the MTS assay (*n* = 3). There was an increase in HK proliferation in the groups ranging from 0 to 1.25 μg/mL concentration. The values are expressed as the mean ± SD and values containing asterisks differ significantly from the control group as shown by one-way analysis of variance (ANOVA, Systat Software, Inc.) (* *p* < 0.05).

AAPE is a component of ADSC-CM, cell culture medium for ADSC. Since AAPE has the effect of the cell growth, we first examined the effect of AAPE on HK proliferation. There was a significant increase in HK proliferation in the experimental groups after the treatment of AAPE compared to the control group (*n* = 3, *p* < 0.05) (Figure 1). However, this increase was observed in the range of 0 to 1.25 μg/mL concentration. The effect was decreased in the groups with concentrations of AAPE exceeding 1.25 μg/mL. This suggests that although AAPE stimulates HK proliferation, this prolific effect occurs only up to certain AAPE concentrations.

### 2.2. DNA Chip Analysis

**Figure 2:**
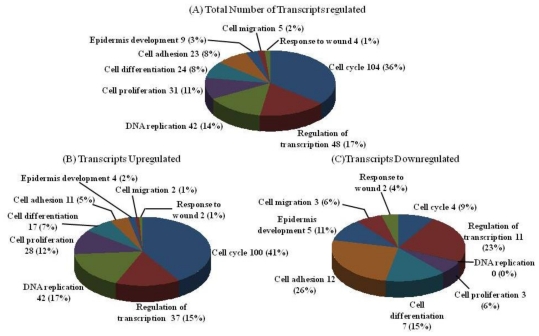
DNA chip analysis. Functional classes of differentially regulated genes in keratinocyte incubated with Advanced Adipose-Derived Stem cell Protein Extract (AAPE). Regulated genes were grouped into nine functional categories and graphed as a percentage of the total, based on their GeneGo designation. 290 genes were differentially regulated based on analysis of the array data (**A**). Of the regulated genes, 243 were up-regulated (**B**) and 53 were down-regulated (**C**). A number of down-regulated genes (12) are associated with cell adhesion; none of the genes in this category were up-regulated. The DNA replication-related transcript category contained 0 down-regulated genes and 42 up-regulated genes. In the cell cycle category, a notable difference in the number of transcripts down-regulated (4) and up-regulated (100) related to cell cycle was observed.

In order to address the gene alterations of the keratinocyte on AAPE, we compared the panel of transcripts whose expression was altered in AAPE-treated keratinocytes compared to AAPE-untreated keratinocytes. We screened DNA chip arrays using RNA isolated from keratinocytes. Our results demonstrate that AAPE in keratinocytes (*p* < 0.05) affected expression of 290 identified transcripts regulated minimally by greater than or equal to a 2-fold change. The identified transcripts were associated with nine functional classes (Figure 2A). Of the identified regulated genes, 243 were up-regulated (Figure 2B) and 53 were down-regulated (Figure 2C). Of the regulated genes, a notable fraction is known to affect cell proliferation and/or cell cycle.

### 2.3. AAPE Stimulates Wounding Healing Cell Migration via ROCK Pathway

**Figure 3:**
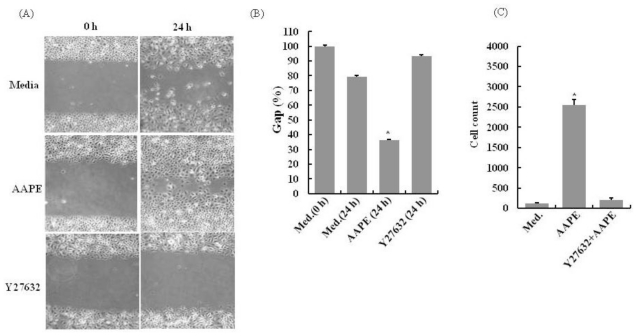
Scratch wound healing assay (*n* = 3) and transwell migration assay (*n* = 3) of keratinocyte in response to AAPE. (**A**) Cells were cultured in keratinocyte culture medium and wound line was created by microtip. After then, cells were incubated with AAPE (1.22 μg/mL) for 24 h. After 24 h, the width of the gaps made by scratching decreased more in the AAPE administered group (AAPE) than in the control group (Medium) or Y27632 treated group (Y27632), Original magnification × 100. (**B**) The percentage signifies the remnant gap size 24 h after making scratches, compared to the initial gap size. The gap width decreased more in the AAPE treated group than in the control group (*n* = 5). (**C**) Chemotactic migration of keratinocytes toward AAPE. Chemotactic activity was determined by constant-period counting using a flow cytometer and is shown as mean ± SD by one-way ANOVA’s t-test. Keratinocytes exhibit typical chemotaxis toward AAPE (1.22 μg/mL), and keratinocyte chemotaxis is dependent on ROCK activity (Y27632+AAPE). Values are expressed as the mean ± SD and asterisk values are significant compared to the control group by one way ANOVA’s test (* *p* < 0.05).

An early event in the process of wound repair is the migration of keratinocytes from wound edges into the wounded area, which is critical for timely healing [9]. The cell scratch assay was used to study the effects of AAPE on HK wound healing. There was a significant decrease in the wound line width in the experimental groups exposed to AAPE compared to the control group (*n* = 4, *p* < 0.05) (Figure 3A,B). This suggests that HK migration had occurred more rapidly in the group exposed to AAPE, indicating that AAPE stimulates HK migration. HK exhibited clear chemotactic migration toward AAPE. RhoA-ROCK signaling has been shown to be involved in the regulation of cell migration [10] including immune cells. To test whether AAPE-enhanced HK migration is involved in those signaling pathways, we further examined the effects of AAPE on HK migration in the presence of specific pathway inhibitors using *in vitro* Transwell system. Y-27632, specific inhibitor of ROCK, inhibited the chemotaxis (*n* = 3, *p* < 0.05) (Figure 3C). Therefore, ROCK activity is required for the proper chemotactic migration of HKs. These findings support the notion that ROCK signaling regulates the efficiency of HK migration.

### 2.4. AAPE Augments Stress Fiber Formation in HK

**Figure 4:**
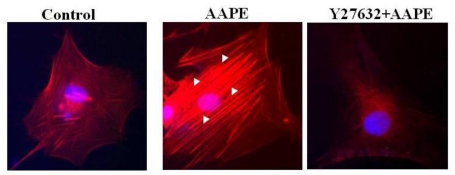
Inhibition of ROCK prevents AAPE-induced actin stress fiber formation. HK was left untreated or challenged for 1 h with AAPE (1.22 μg/mL) in the absence or presence of 10 μM Y27632. The cells were then fixed, permeabilized, and stained with rhodamine phalloidin to visualize the actin stress fibers by fluorescence microscopy. The results are representative of three experiments.

Stress fibers are composed of bundles of approximately 10–30 actin filaments [11] held together by the actin-crosslinking protein such as fascin, espin and filamin [12–15]. This serve as a cross-linker between the towing and trailing adhesions, and their organization reflects the direction of the traction force. In motile fibroblasts, ventral stress fibers are oriented parallel to the axis of locomotion [11], which suggests that force generated by contraction of these structures could drive tail retraction. Therefore, these structures provide mechanical contractile force for cell migration. Because stress fiber formation is a cell response characteristic of keratinocyte [15] and fibroblast [16] migration, we investigated whether stress fiber formation is induced by AAPE and that ROCK signaling is involved in stress fiber formation leading to the control of actin cytoskeleton reorganization [15]. Stress fiber formation was markedly enhanced by the stimulation of AAPE (Figure 4) in HK, whereas the stimulation of cells by Y27632, a ROCK inhibitor, completely abolished it (Figure 4). We therefore propose that the induction of stress fiber via stimulation with AAPE requires the ROCK pathway, ultimately leading to the facilitation of cell migration.

### 2.5. RhoA-ROCK Pathway Is Involved in Actin Stress Fiber Formation in HK

**Figure 5:**
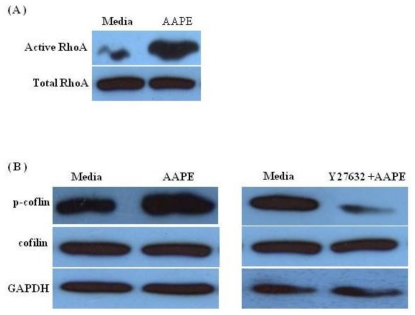
RhoA-ROCK activity is associated with phosphorylation of cofilin in HK. RhoA pull down assay and Western blot were performed for detection of active RhoA (**A**) and AAPE, Y27632+AAPE and control HK were assessed by Western blot for cofilin and p-cofilin (**B**). The Western blot membrane was normalized for GAPDH to control loading.

Considerable evidence indicates that RhoA-ROCK pathway signals the reorganization of the actin cytoskeleton, which induces the formation of stress fibers [17,18]. To address the possibility that the stress fiber alteration of AAPE treated HK is involved in RhoA-ROCK signaling, we checked the level of RhoA-GDP/GTP exchange activity with HK lysates. Using the cell lysate, the exchange activity was assessed by a nucleotide exchange reaction of RhoA-GDP, followed by RBD (Rhodekin-binding domain)-GST-mediated pull-down detection of RhoA-GTP. As seen in Figure 5A, when HK was cultured with AAPE, the exchange activity was markedly increased. An important effector of RhoA is ROCK, which, together with other kinases, contributes to the phosphorylation of cofilin, which is involved in remodeling of the actin cytoskeleton. To test whether AAPE and Y27632 combined with AAPE in HK affects phosphorylation of cofilin, we performed Western blot analysis of HK lysate. In the presence of AAPE, phosphorylated cofilin was increased, whereas, the amount of inactive, phosphorylated cofilin was reduced in Y27632+AAPE sample (Figure 5B). These results revealed that stress fiber formation was involved in RhoA-ROCK mediated cytoskeletal remodeling in HK.

### 2.6. Protein Profile of Conditioned Medium, AAPE from Naïve Primary ADSC Cultures

**Figure 6:**
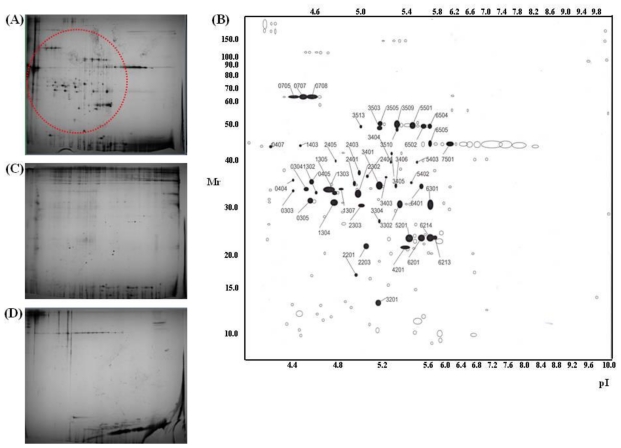
2-D gel analysis of protein samples. Silver-stained 2-D maps of AAPE (**A,B**), collagen (**C**) and fibronetin (**D**). Red circle (**A**) and numbered spots (**B**) indicate the proteins excised for identification by MS.

To assess the component of protein pools of AAPE, we carried out 2-D gel electrophoresis and MALDI-TOF analysis. Collagen and fibronetin in extracellular matrix (ECM) compartments which play an important role in skin regeneration in comparison with controls were also analyzed by 2-D gel electrophoresis. After silver staining of 2-D gels, protein spots were digested in-gel with trypsin. Extracted peptides corresponding to gel slices were analyzed by MALDI-TOF for protein mass finger printing. Preliminary separations using AAPE samples revealed that various spots between ~15 kDa and ~70 kDa different from those of collagen (Figure 6C) and fibronetin (Figure 6D) could be detected by 2-D page (Figure 6A,B). Of the selected proteins (50) 48 proteins were identified but 2 proteins could not be determined (Table 1). The identification of selected protein was divided by 64% collagen components and 30% non-collagen components (Table 1). 40.6% α-1 type VI collagen, 18.75% type I collagen, 18.75% pro α-1 type I collagen, 15.6% α-1 type III procollagen, and 6.25% α-2 type VI collagen were distributed in the collagen category, respectively. AAPE also contained the connective tissue growth factor (CTGF) which is an ECM-associated heparin-binding protein and involved in granulation tissue formation, re-epithelisation, matrix formation and remodeling [19]. In addition, IL-6 which has been shown to be a proliferative effect [20,21] and migration [22] on keratinocytes was included in AAPE. Moreover, selected spots contained plasminogen activator inhibitor type-1 (PAI-1, SERPINE1). Cutaneous tissue injury initiates the activation of PAI-1. Such PAI-1 significantly stimulates the directional migration of keratinocyte during the real-time of monolayer scrape-injury repair. Together, these results suggest that AAPE functions as the regenerator of cutaneous tissue healing.

### 2.7. Antibody Array

**Figure 7:**
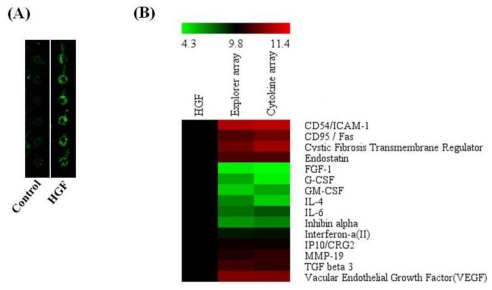
Antibody array of AAPE. (**A**) AAPE was subjected to antibody array. Portions of the array illustrating the differential expression of proteins between control and HGF antibody are shown. Each panel contains six replicates of a specific antibody-protein reaction. (**B**) Quantitation of the average signal for each protein between samples. Heat map were analyzed by Genowiz 4.0^TM^ as described in Materials and Methods. The median streptavidin–Cy3 fluorescence from all microsphere subsets was exported. The heat map shows antibody reactivity intensity (*i.e.*, values above HGF) as red pixels and (values below HGF) as green pixels.

Although the 2-D gel analysis of AAPE supplied some protein information, we thought that more soluble proteins including cytokines exist in AAPE. Thus, an explorer antibody array chip coated with 656 antibodies was conducted to identify proteins in AAPE (Figure 7). Protein level measurements were clustered by the ratio of 4.57% hormone, 11.7% cytokine, 8.99% cell cycle, 11.33% apoptosis, 19.21% angiogenesis, 8.69% stem cell, and 24.70% signal transduction level by comparing the signal intensity of soluble protein expression in AAPE to a negative empty control (data not shown). Furthermore, a cytokine-focused antibody array with 77 antibodies was conducted to identify proteins that may be involved in mediating the effect of AAPE on proliferation, migration or regeneration (Figure 7) by comparison with explorer antibody array data. *p* value < 0.05 was considered significant. Analysis of the array results revealed the expression of several proteins different from that of 2-D analysis including HGF, FGF-1, G-CSF, GM-CSF, IL-6, VEGF, and TGF-β3 (Figure 7B). HGF is composed of 60 kDa alpha-chain and a 34 kDa beta-chain. It induced keratinocyte *in vitro* scratch-wound healing in a concentration-dependent manner [23]. IL-6 has a proliferative and migration effect on mouse keratinocytes [20,22]. GM-CSF also accelerates wound healing: Stimulation of keratinocyte proliferation, granulation tissue formation, and vascularization [24]. TGF-β3 promotes wound healing by recruiting fibroblast to the wound site. These proteins provide evidences of the usefulness of AAPE in the skin regeneration.

### 2.8. Discussion

Oxygen deficiency, *i.e.*, hypoxia, may impair cell biological action. However, cellular functions to hypoxic stress are highly dependent in cell type, position and micro-environment. ADSCs are thought to reside in hypoxic regions surrounded with various tissues in complicated 3-dimensional space of the human body. When ADSCs are cultured under hypoxic conditions *in vitro*, their proliferative and self-renewal capacities are significantly improved [25] and hypoxia enhanced the secretion of certain growth factors [26]. We investigated the ability of ADSC secretomes to promote HK regeneration. ADSC-CM stimulates the growth of multiple cell types via autocrine or paracrine action. ADSCs were isolated from adipose tissue samples via elective liposuction and were cultured in bulk cell factories by our group. So, we called the conditioned-medium of ADSC an AAPE. Skin wounds are a complex process having combined efforts of multiple types and lineages of skin cells, ECMs, and soluble GFs. Inflammation, reepithelialization, ECM reorganization and tissue remodeling are proposed sequential events to repair skin wounds [27,28]. Keratinocytes activated during wound healing release growth factors and various cytokines that stimulate fibroblasts and endothelial cells, initiate the influx of immune cells, and produce systemic effects [29,30]. They are also the source of extracellular matrix proteins, and adhesive molecules. This study examined whether or not locally applied AAPE can accelerate the wound-healing process *in vitro* system. The proliferation of human primary epidermal keratinocyte, which is cell lines derived from the major cell type present in skin, was increased by AAPE treatment. The triggering of keratinocyte migration by various attractants involves complicated signaling, although the overall picture of it remains incomplete. AAPE are thought to trigger the sequential signaling events of the ROCK pathway and to induce the stress fiber of keratinocytes that are able to migrate efficiently (Figure 4). Therefore, these findings support the notion that ROCK signaling participate in the efficiency of keratinocyte migration. Cell mobility is generally described as a periodic process between alternating phases of protrusion and adhesion. Adhesion provides the traction point required for generating pulling forces, and the cell moves forward direction by the tension generated by contraction of the cell body and retraction of the tail [31]. Because stress fiber provides contractile force derived from the contractile nature, cell migration is dependent on rearrangement of cell cytoskeleton, predominantly actin filaments. Thus, the stress fiber was observed during cell migration, suggesting that this structure might be important for efficient cell motility. AAPE owes its action in the regeneration ability to several ADSC secretomes identified by proteome analysis via a 2-D gel analysis and an antibody array. Hepatocyte growth factor (HGF) stimulates migration of neutrophils, monocytes and mast cells into wounded areas [32] and promotes secretion of pro-angiogenesis factors [33]. HaCaT cells are stimulated to proliferate by HGF [34] and scratch assays were performed in the presence and absence of HGF treatment to assess the response of HaCaT cells to HGF. In response to HGF, cells moved into the wound and did not scatter [35]. PAI-1, a member of the serine protease inhibitor (serpin) superfamily, is known to have a critical role in the fibrinolytic system [36]. In addition to the fibrinolytic system, there is evidence supporting the involvement of PAI-1 in several other cell-biological phenomena, including wound healing [37–39]. Addition of active recombinant PAI-1 to wounded wild-type keratinocyte monolayers significantly stimulated directional movement above basal levels [40]. IL-6-deficient transgenic mice (IL-6 KO) display that IL-6 could influence wound healing by inducing keratinocyte migration through the production of a soluble fibroblast-derived factor. IL-6 has also a proliferative effect [20] on mouse keratinocytes. CTGF expression is increased after injury and is involved in reepithelialization, and matrix formation and remodeling in human dermal fibroblast [19]. GM-CSF protein level has been shown to be increased in the epidermis in wounded skin [24]. It is very important during the inflammatory reaction of wound healing enhancing neutrophils’ function at the wound site [24]. In addition, Blessing *et. al* reported GM-CSF increased keratinocyte proliferation and thus enhanced reepithelialization [24]. TGF-β3 has also been shown to be involved in wound healing. *In vivo* studies have shown that TGF-β3 promotes wound healing by facilitating keratinocyte migration [41]. However, AAPE contained pigment epithelium-derived factor (PEDF), a 50 kDa glyco-protein and a member of the serine protease inhibitor gene family. PEDF inhibits proliferation of HaCaT cells and induces S phase accumulation, and induces a significant decrease of the wound healing of HaCaT cells [42]. The close correlation between scratch wound and cell migration data suggest that PEDF can inhibit migration of HaCaT cells. AAPE from hypoxic cultures of ADSCs increased HK proliferation, migration, and stress fiber formation providing cell motility. AAPE significantly reduced the wound size. In our proteomic analysis, AAPEs contained several collagens, fibronectin, growth factors, and cytokines. These data suggest that ADSC secretomes are well suitable for dermal regeneration. However, these results might vary when using other extracts prepared from the pooled ADSCs of different donors. In a highly concerted biological process of dermal regeneration, HKs interact with surrounding cells such as fibroblasts, fat cells and immune cells. Mainly with secreted growth factors, cytokines or ECM proteins, AAPE contribute to enhance wound healing and skin regeneration via HKs. This study supplies the clues of the regenerative effect of the AAPE. For the proper application of soluble factors of AAPE for skin biology, more studies about the action mechanisms of AAPE on skin and standard application method of AAPE are necessary.

## 3. Materials and Methods

### 3.1. Stem Cell Culture and AAPE Production

Human subcutaneous adipose tissues were obtained from 23 healthy women with informed consents as approved by the institutional review boards by medical liposuction. The age distribution of the patients ranged from 26 to 51 years, with a mean of 36.7 years and about 67 kg. The adipose tissues were exposed to collagenase (final 0.075% type II collagenase, Sigma-Aldrich, St. Louis, MO, USA) for 30 min at culture temperature, followed by centrifugation at 400 g for 10 min, washed and resuspended in PBS. The stromal cell fraction was filtered through a 70 μm cell strainer (BD Biosciences, San Jose, CA, USA). Using Histopaque-1077 (Sigma-Aldrich, St. Louis, MO, USA), ADSCs were isolated from the filtrate, then cultured at 37 °C, 5% CO_2_ in DMEM containing 10% FBS. Characteristic expressions of stem cell-related surface markers were confirmed by flow cytometry [9,10]. ADSCs expressed CD73, CD90 and CD105, and were lacking in CD34 and CD49d. Adipogenic, osteogenic, and chondrogenic differentiation was also checked by the conventional method [43,44]. After the isolation of ADSC from patients, cells were pooled. ADSCs were cultured and expanded in normal control medium, and used for the experiments at passages 4. Cells were finally frozen in aliquots using CellFreezer^TM^ (Genenmed, Seoul, Korea) for the future. To produce a ADSC-CM (AAPE^TM^), a frozen vial containing 1 × 10^6^ cells were launched onto culture medium containing 10% FBS. After repeating subcultures to reach 5 × 10^8^ cells, the expanded ADSCs were introduced into CellFactory^TM^ CF10 (Nunc, Rochester, NY, USA) in DMEM/F12 serum-free medium (Welgene, Taegu, Korea). Cultures were conducted under a hypoxia by providing 2% O_2_ using N_2_ gas supply in a humidified multichannel incubator during 2 weeks. The conditioned media were collected and micro-filtered, followed by quantitated total protein production. Finally, for fresh use, 4 mL vials containing equal protein concentration were freeze-dried as a single lot sample preparation of AAPE (Prostemics Research Institute, Sungnam, Korea) for this study.

### 3.2. Cell Culture

Normal HKs were purchased from ATCC cell bank (ATCC# PCS-200-011**)**. HKs were cultured in serum-free keratinocyte media (ATCC PCS-200-030) with epidermal growth factor at concentrations of 0.2% (v/v) of bovine pituitary extract, 5 g/mL bovine insulin, 0.18 g/mL hydrocortisone, 5 g/mL bovine transferring, 0.2 ng/mL human epidermal growth factor (EGF) at 37 °C in a 5% CO_2_ humidified atmosphere.

### 3.3. Cell Proliferation

Cell viability and proliferation was determined using CellTiter 96 Aqueous One Solution Reagent (Promega, Madison, WI, USA). Briefly, cells (2 × 10^5^) were placed in 96-well plastic culture plates and incubated at 37 °C in 5% CO_2_ for 24 h, at which point 100 μL of 0.5 mg/mL MTS solution was added to each well and incubated for 4 h at 37 °C. Formazan absorbance was read at 490 nm using a plate reader.

### 3.4. DNA Chip Analysis

For this analysis with GeneChip Human Gene 1.0 ST array (Affymetrix, Santa Clara, CA, USA) containing 28,869 gene-level probe set, total cellular RNA was isolated from keratinocyte incubated with AAPE (1.25 ug/mL) for 24 h using Trizol reagent (Invitrogen, Foster, CA, USA) according to manufacturer’s directions. The RNA samples were submitted to the Advanced Medical Technology Center for Diagnosis & Prediction (School of Medicine, Kyungpook National University, Taegu, Korea) for array preparation and analysis. The signal intensity of the gene expression level was calculated by Expression Console software, Version 1.1 (Affymetrix). The list was filtered first for the absent genes, secondly for a fold change cutoff of 2, and thirdly for *p* value of ≤0.05 using Welch’s *t*-test.

### 3.5. *In vitro* Migration Assay

The migration assay reported by Li *et al.* was modified [45]. HKs were cultured in a 6-well culture plate (2.5 × 10^5^ per well) for 24 h until 90% of the base was filled. Wound line was created by scratching the plates with a 10 μL micropipette tip. Two groups of culture fluid with AAPE (*i.e.*, 0, and 1.22 μg/mL) were made after removing the floating cells with PBS, which was then cultured for 24 h with keratinocyte media without growth factors. Mitomycin C (10 μg/mL) was added to the culture solution to avoid cell proliferation. The gap of the control group was taken as 100% to assess cellular migration, and compared with that of the experimental group. We prepared five different plates for each group, totally prepared 15 plates. Just one wound line was made in each plate. The average width of the gaps was calculated from the image taken with a microscope at five different sites from each wound line (n = 5). In total, 25 sites were measured in each group.

### 3.6. Chemotaxis Assay

The chemotaxis assay was performed using Transwell chambers (6.5 mm diam, 5 mm pore size, Costar). 1 × 10^5^ cells suspended in 100 μL of medium were placed into the top chamber, and 600 μL of medium containing AAPE (1.22 μg/mL) was added to the bottom well. Alternatively, cells were pretreated with 10 μM Y-27632 (Calbiochem, Luzern, Switzerland) in keratinocyte dermal cell basal media for 30 min at 37 °C. After 4 h of chemotaxis, cells in the bottom well were collected and the cell number was counted using a FACScalibur flow cytometer (Becton Dickinson, NJ, USA).

### 3.7. Fluorescence Microscopy for Stress Fiber Formation

HKs on collagen coated chamber slides (Lab-Tek, Nalge Nunc Int. Naperville, IL, USA) were cultured in growth factor free keratinocyte media for 12 h. Alternatively, cells were pretreated with 10 μM Y-27632 (Calbiochem, Luzern, Switzerland) in HK for 30 min at 37 °C. The cells were treated with the AAPE (1.25 μg/mL) for 24 h, then fixed in formalin and treated with ice-cold methanol for 10 min. The cells were then stained with rhodamine phalloidin and observed by fluorescence microscopy.

### 3.8. RhoA Pull Down

More than 3 × 10^8^ cells were lysed with 2 mL RIPA buffer. The amount of Rho-GTP in the reaction solution was measured by a pull-down method based on specific binding to Rhotekin-RBD followed by Western blotting using anti-Rho antibody (Rho activation assay biochem kit; BK306; Cyoskeleton, Inc.). The relative amount of active Rho compared with that in the control was calculated by measuring the band density of Rho and normalized total RhoA density

### 3.9. Western Blot

HKs pretreated with AAPE (1.25 μg/mL), 10 μM Y27632 were lysed in 5× SDS sample buffer. After the samples were boiled, equal amounts of total lysates were separated by SDS-PAGE and transferred onto polyvinylidene difluoride membranes. The membranes were soaked in a blocking solution (5% skim milk and 0.2% Tween 20-PBS) for 1 h, and then incubated with anti-p-cofilin, and anti-cofilin antibodies for 1 h. GADPH (R&D Systems, Wiesbaden, Germany) was used for loading control. After being washed with Tween 20-PBS, membranes were incubated with appropriate HRP-conjugated secondary antibodies for 1 h. Specific bands were visualized by an ECL method (ECL^+^; Amersham Biosciences).

### 3.10. Proteome Analysis

AAPE soluble fraction was used for two-dimensional gel electrophoresis. Protein loading was normalized by Bradford assay [46]. IPG dry strips were equilibrated for 12–16 h with 7 M urea, 2 M thiourea containing 2% 3-[(3-cholamidopropy) dimethyammonio]-1-propanesulfonate (CHAPS), 1% dithiothreitol (DTT), 1% pharmalyte and respectively loaded with 200 ug of sample. Isoelectric focusing (IEF) was performed at 20 °C using a Multiphor II electrophoresis unit and EPS 3500 XL power supply (Amersham Biosciences, Piscataway, NJ, USA) following manufacturer’s instruction. SDS-PAGE was performed using Hoefer DALT 2D system (Amersham Biosciences) following manufacturer’s instruction. 2D gels were run at 20 °C for 1700 Vh. And then 2D gels were silver stained as described by Oakley *et al.* [47]. Quantitative analysis of digitized images was carried out using the PDQuest (version 7.0, BioRad, Hercules, CA, USA) software according to the protocols provided by the manufacturer. For protein identification by peptide mass fingerprinting, protein spots were excised, digested with trypsin (Promega, Madison, WI, USA), mixed with α-cyano-4-hydroxycinnamic acid in 50% acetonitrile/0.1% TFA, and subjected to MALDI-TOF analysis (Ettan MALDI-TOF Pro, Amersham Biosciences, Piscataway, NJ, USA) as described Fernandez J *et al.* [48]. Spectra were collected from 350 shots per spectrum over m/z range 600–3000 and calibrated by two point internal calibration using Trypsin auto-digestion peaks (m/z 842.5099, 2211.1046). Peak list was generated using Ettan MALDI-TOF Pro Evaluation Module (version 2.0.16). Threshold used for peak-picking was as follows: 5000 for minimum resolution of monoisotopic mass, 2.5 for S/N. The search program MASCOT, developed by The Matrixscience (http://www.matrixscience.com/), was used for protein identification by peptide mass fingerprinting.

### 3.11. Antibody Array

The freeze dried protein sample was submitted to the Ebiogen (Kyung Hee business center, Kyung Hee University, Seoul, Korea) for array preparation and analysis. The explorer antibody microarray slide (Fullmoon biosystems, Sunnyvale, CA, USA) with 656 antibodies and the cytokine-focused microarray slide with 77 antibodies were treated 30 mL of blocking solution and incubated on shaker for 45 minutes at room temperature. After blocking, the slide was washed 10 times with 45 mL of distilled water. The labeled sample was mixed in 5.3 mL of coupling solution. The blocked array slide was incubated with coupling mixture on shaker at 35 rpm for 2 h at room temperature into coupling dish. After coupling, the slide was washed 2 times with 30 mL of washing solution. The 30 μL of 0.5 mg/mL Cy3-streptavidin (GE Healthcare, Chalfont St. Giles, UK) was mixed in 30 mL of detection buffer. The slide scanning was performed using Revolution^TM^ 4200 microarray scanner (Vidar Systems, Herndon, VA, USA) and ArraySifter Express 1.3 (Vidar Systems). The slide was scanned at 10 μm resolution, optimal laser power and PMT. After got the scan image, it was grided and quantified with ArraySifter Express 1.3. The numeric data were analyzed using Genowiz 4.0^TM^ (Ocimum Biosolutions, India). After analyzing, the data about protein information were annotated using UniProt DB.

## 4. Conclusions

The present research reports on the beneficial effects of AAPE on skin regeneration. AAPE activates keratinocyte proliferation and migration. Although the mechanism of how AAPE promotes skin regeneration is unclear, AAPE contained various growth factors when examined via proteome analyses such as, 2-D gel assay, MALDI-TOF analysis and antibody array, confirming it to be a great resource for skin regeneration. These results highlight the potential of topical application of AAPE in the treatment of skin regeneration.

## Figures and Tables

**Table 1:** Proteins identified from spots by Mascot search of the NCBInr database (Collagen).

Trypsin spot	Protein name	Accession #	Protein score	Pep. count	Position	Sequence coverage
303	Type VI collagen, alpha-2, isoform 2C2a’	NP_478055	86	9	96–393	12%
304	Human pro alpha 1(I) collagen	CAA29605	138	7	9–196	45%
305	Type VI collagen, alpha-1 chain	CAA33888	210	11	52–256	50%
404	Type VI collagen, alpha-1 C-terminal domain	CAA33888	155	8	69–211	39%
405	Type VI collagen, alpha-1 C-terminal domain	CAA33888	162	9	52–256	41%
1302	Type VI collagen, alpha 2, isoform	EAX09316	103	9	96–411	12%
1303	Human pro alpha 1(I) collagen	CAA29605	142	11	9–196	48%
1304	Type VI collagen, alpha-1 C-terminal	CAA33888	288	16	28–256	67%
1305	Carboxy-propeptide of alpha 1 (III) procollagen	CAA25879	150	11	1–211	45%
1307	Carboxy-propeptide of alpha 1 (III) procollagen	CAA25879	41	3	46–52	18%
2302	Carboxy-propeptide of alpha 1 (III) procollagen	CAA25879	179	13	1–211	43%
2303	Type VI collagen, alpha-1 C-terminal domain	CAA33888	139	8	52–256	30%
2401	Type I collagen	CAA39142	120	9	165–361	25%
2403	Human pro alpha 1(I) collagen	CAA29605	129	7	9–196	45%
2404	Carboxy-propeptide of alpha 1 (III) procollagen	CAA25879	213	13	43–211	47%
3302	Type I collagen	CAA39142	137	12	165–361	29%
3401	Type I collagen	CAA39142	147	12	165–361	29%
3403	Carboxy-propeptide of alpha 1 (III) procollagen	CAA25879	175	12	43–211	44%
3404	Pro alpha 1(I) collagen	CAA29605	85	7	9–171	37%
3405	Pro alpha 1(I) collagen	CAA29605	90	8	9–171	30%
3406	Type I collagen	CAA39142	127	9	165–361	25%
3503	Type VI collagen, alpha-1	CAA33889	114	9	91–431	23%
3505	Type VI collagen, alpha-1	CAA33889	148	12	101–432	27%
3509	Type VI collagen, alpha-1	CAA33889	187	13	91–432	30%
3510	Type VI collagen, alpha-1	CAA33889	94	7	120–431	20%
3513	Type VI collagen, alpha-1	CAA33889	97	8	120–431	21%
5403	Pro alpha 1(I) collagen	CAA29605	133	10	9–196	49%
5501	Type VI collagen, alpha-1	CAA33889	208	14	91–432	31%
6301	Type I collagen	CAA39142	217	16	165–361	41%
6401	Type I collagen	CAA39142	164	14	165–361	29%
6502	Type VI collagen, alpha-1	CAA33889	134	10	120–431	27%
407	Human Plasminogen Activator Inhibitor Type-1	1A7C_A	182	15	1–379	42%
705	Gelatinase A	1CK7_A	185	18	1–631	34%
707	Gelatinase A	1CK7_A	249	23	1–561	47%
708	Gelatinase A	1CK7_A	247	21	1–561	40%
2201	Connective tissue growth factor precursor	NP_001892	77	5	43–152	14%
2203	Interleukin-6 precursor	NP_000591	96	5	59–207	22%
2405	Human procathepsin L	1CJL_A	97	6	18–292	23%
3201	Cadherin 2, isoform	EAX01239	64	6	34–114	8%
4201	Heparan sulfate proteoglycan 2	EWA94994	67	11	181–3588	3%
5201	Prommp-2TIMP-2	1GXD_C	62	4	28–194	19%
6201	SPAG9 protein	AAI06049	70	7	1–461	21%
6213	Prommp-2TIMP	1GXD_C	68	5	28–194	23%
6214	Prommp-2TIMP	1GXD_C	89	7	28–194	30%
6505	Chain A of human PEDF	1IMV_A	141	10	34–391	24%
7501	Chain A of human PEDF	1IMV_A	161	13	34–398	30%
1403	N/I *					
3304	N/I					
5402	N/I					

* : Not determined.
